# How does the sagittal spinal balance of the scoliotic population deviate from the asymptomatic population?

**DOI:** 10.1186/s12891-018-1954-5

**Published:** 2018-02-02

**Authors:** Pan-pan Hu, Miao Yu, Xiao-guang Liu, Zhong-qiang Chen, Zhong-jun Liu

**Affiliations:** Department of Orthopedics, Peking University Third Hospital, 49 North Garden Rd., Haidian District, Beijing, 100191 China

**Keywords:** Sagittal spinal balance, Asymptomatic, Idiopathic scoliosis, Young adults, Roussouly classification

## Abstract

**Background:**

Previously, the sagittal spinal balance in both asymptomatic and scoliotic Caucasian people has been characterized and compared. Very recently, the sagittal spino-pelvic parameters among asymptomatic Chinese adults have been studied, and the results were compared with Caucasian adults, indicating that a difference did exist. Unfortunately, the distribution of sagittal standing posture patterns among the Chinese population has not been characterized in either asymptomatic or scoliotic groups.

**Methods:**

We conducted a radiographic comparison study to define the deviation of sagittal balance in scoliotic patients from that of an asymptomatic population. A total of 126 asymptomatic and 117 idiopathic scoliotic (IS) young adults were recruited. Radiographic data from each subject were reviewed, and sagittal spinopelvic parameters were measured. The Roussouly type was then determined, as well as the relative position of the C7 plumbline with respect to the sacrum and hip axis. Comparison analyses were undertaken between the two different groups.

**Results:**

The IS group had a larger pelvic incidence, pelvic tilt and sacral slope, but a smaller spinal tilt than the asymptomatic group (*P* < 0.05), while other sagittal parameters were similar. The distribution of Roussouly types was similar between the asymptomatic and IS groups, of which 49.2% and 45.3% belonged to Roussouly Type 3, respectively. Asymptomatic males and females had a similar distribution, which was different between the two genders in the IS group (*P* < 0.05), with more females possessing a neutral sagittal standing posture. In addition, more IS subjects had forward displacement of the C7 plumbline than asymptomatic ones (P < 0.05), while there was no difference between the two genders in either group.

**Conclusions:**

Although sagittal pelvic parameters were greater in the IS population, their sagittal spinal balance was maintained and there was no sagittal standing posture pattern correlated with IS. The occurrence of anterior displacement of the C7 plumbline was more common in IS patients than asymptomatic adults, but did not appear to be correlated with gender in both populations.

## Background

**Fig. 1:**
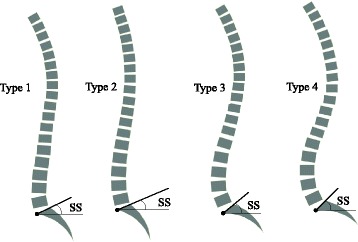
Representative drawings of Roussouly types. **Type 1** Sacral slope (SS) < 35°, apex of lumbar lordosis (LL) at middle L5, the spine is hypolordotic and relatively normokyphotic; **Type 2** SS < 35°, apex of LL at base L4, the spine is hypolordotic and hypokyphotic; **Type 3** 35° < SS < 45°, apex of LL at middle L4, the spine is well balanced; **Type 4** SS > 45°, apex of LL at base L3, the spine is hyperlordotic and hyperkyphotic

Recently, the study of sagittal spinal balance in different populations has gained importance ever since Roussouly et al. [1] proposed a four-type classification in a study of 160 normal European adults. This novel classification is mainly determined by sacral and lumbar sagittal parameters such as sacral slope (SS), lumbar lordosis (LL), the number of lumbar vertebrae in LL, inflection point and lumbar tilt (Fig. 1). However, there is a lack of literature that comprehensively analyses and compares the deviation of sagittal standing posture patterns in the scoliotic population with their normal counterparts, though previous publications have studied the difference of some sagittal spinopelvic parameters between the two populations [2, 3]. Upasani et al. [2] compared the measurements of sagittal spinopelvic parameters between scoliotic and asymptomatic adolescent subjects and found that scoliotic patients had greater pelvic incidence (PI) and pelvic tilt (PT) values than the asymptomatic population. They also found that patients with primary thoracic scoliosis had a lower thoracic kyphosis (TK) and a larger SS than the controls, whereas patients with a primary thoracolumbar scoliosis had relatively normal TK, LL and SS values. Qiu et al. [3] compared the values of sagittal spinopelvic parameters between adolescent girls with idiopathic thoracic scoliosis and asymptomatic girls and found that TK in the former group was lower, which supported the findings of Upasani et al. [2]. Other radiographic studies in this field also reported similar results [4, 5]. However, there is no published comparative study that specifically explored the difference of the whole sagittal spinal alignments and the distribution of standing posture patterns in scoliotic patients from the norm.

Hu et al. [6] reported that 42.4% of scoliotic adolescents belonged to Roussouly Type 3. This proportion is considerably high when compared with that of the general population reported by previous publications [1, 7–9]. Thus, a question was raised regarding how the sagittal standing posture patterns of the scoliotic population deviated from the norm. Aiming to illuminate the above question, this study was designed to describe and compare the whole sagittal spinal balance and the distribution of their patterns in asymptomatic and scoliotic young adults.

## Methods

### Materials

Young adults with idiopathic scoliosis (IS) who visited our hospital from Jun 2006 to Dec 2013 were consecutively reviewed and screened, regardless of whether they underwent correction surgery or not. The inclusion criteria were as follows: (1) aged 18 to 34 years old; (2) diagnosed with IS with no other pathological conditions; (3) having structural curve(s) with Cobb angle(s) of 25 degrees or more on side-bending films; (4) having an entire set of films (namely, full-length posteroanterior, side-bending and lateral X-rays) taken at our hospital; (5) no previous medical intervention on spine, pelvis or low extremities. An age-matched enrolment of normal counterparts was taken among medical students, young doctors, nurses and other employees in our hospital. Their medical histories were examined, and individuals were excluded if they had (1) previous trauma or medical intervention on the spine, pelvis or low extremities; (2) any symptom suggestive of spinal or other orthopaedic diseases; (3) any radiographic anomaly during the process of imaging review, such as scoliosis, spondylolisthesis, spondylolysis, severely wedged vertebrae beyond the normal range of variation and leg discrepancy. This study was permitted by the Institutional Review Board, and informed consent was obtained from all individual participants included in the study.

### Methods

#### Imaging protocols and parameters to measure

All X-rays included in the study were taken before brace and surgical intervention. To facilitate the comparison, an identical radiographic protocol, which was consistently adopted in our hospital, was applied to both groups. No side-bending films were used for normal subjects. Full-length posteroanterior (PA), side-bending (only for IS subjects) and lateral X-rays of the spine-pelvis complex were taken. When taking lateral films, subjects stood in an erect and comfortable position, with arms flexed 45 to 60 degrees and hands placed on adjustable supports with horizontal gaze. Exposures were taken from the base of the skull to the proximal femora. The distance from the radiographic source to the films was maintained at 180 cm, and the edges of the films were squared with respect to the horizontal and vertical axes separately. The films were digitized with a commercially available optical scanner (XR 650, GE, USA). All morphologic data were archived via Picture Archiving and Communication Systems (PACS, GE, USA). Then, the data were retrieved and measured through PACS in diagonal 20-in. screens with a resolution of 75 dpi.

**Fig. 2:**
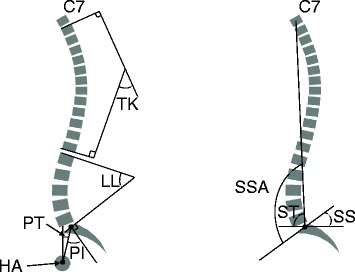
Measurements of sagittal spinopelvic parameters. TK, thoracic kyphosis; LL, lumbar lordosis; PI, pelvic incidence; PT, pelvic tilt; SS, sacral slope; SSA, spinosacral angle; ST, spinal tilt. HA refers to hip axis

First, bending films of IS subjects were retrieved for the confirmation of previous diagnoses, according to Lenke’s definition [10]. Sagittal parameters to be measured included thoracic kyphosis (TK), the angle subtended by the T1 superior endplate and T12 inferior endplate; lumbar lordosis (LL), the angle subtended by the L1 and S1 superior endplates; pelvic incidence (PI), the angle subtended by the line connecting the hip axis (HA, the midpoint of the line connecting bicoxofemoral centres) to the midpoint of the upper sacral endplate and the line perpendicular to the upper sacral endplate; pelvic tilt (PT), the angle subtended by the vertical line and the line connecting the HA to the midpoint of the upper sacral endplate (Fig. 2); sacral slope (SS), the angle subtended by the horizontal line and upper sacral endplate, and spinosacral angle (SSA), the angle subtended by the upper sacral endplate and the line from the centre of the C7 vertebral body to the midpoint of the upper sacral endplate. Moreover, spinal tilt (ST), the angle subtended by the line connecting the horizontal line and the centre of the C7 vertebral body to the midpoint of the upper sacral endplate, was calculated by the following formula: ST = SSA - SS (Fig. 2). On PA films, any significant curve was re-measured for IS subjects, while for normal subjects, any curve more than 10° was marked for exclusion. All values were measured two times by a reviewer, and the average results were obtained.

**Fig. 3:**
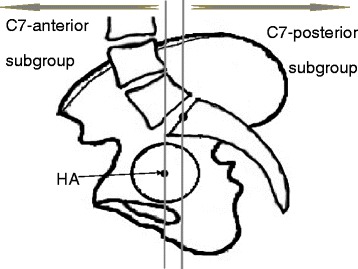
Determination of C7-subgroups. If C7 plumbline was ahead of HA and midpoint of upper sacral endplate, the subject belonged to C7-anterior subgroup. Otherwise, the subject was placed in C7-posterior subgroup

All subjects were categorized by Roussouly classification [1] according to their PI, SS, PT, thoracic and lumbar alignments (Fig. 1). In addition, the relative position of the C7 plumbline with respect to the HA and the midpoint of the sacral upper endplate was determined and divided into two subgroups [4], classified as either C7-anterior subgroup if the C7 plumbline was ahead of both the midpoint of the upper sacral endplate and the HA, or C7-posterior subgroup (Fig. 3).

### Statistical analysis

Statistical analyses were conducted using IBM SPSS Statistics for Windows Version 20 (IBM Corp., Armonk, New York, USA). The precision of measurement was determined using a technical error of measurement (TEM), and the coefficient of reliability (R) was calculated [11]. Descriptive statistics were provided as the mean ± standard deviation (SD) and 95% confidence interval (95%CI). Two-tailed unpaired Student’s t-test and two-tailed Pearson’s χ^2^ test (or Fisher’s exact test if indicated) were utilized to compare between different groups. Statistical significance was set at 0.05.

## Results

**Fig. 4:**
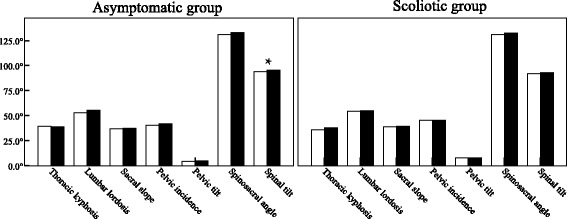
Comparison of sagittal parameters between males (blank bars) and females (black bars) in both groups, using two-tailed unpaired Student’s t-test. *Statistically significant at *P* < 0.05 (*P* = 0.021, *t* = − 2.334, *df* = 124)

In all, 126 asymptomatic and 117 IS subjects were recruited, with an average age of 26.4 ± 3.7 and 26.3 ± 4.7 years, respectively (Table 1). The Cobb angles of coronal curves for IS adults are presented in Table 2. All sagittal parameters were normally distributed, and the reliability (R values) for all parameters was above 96%, ranging from 96.88% to 99.98%. The PI for the normal and IS groups was 41.2° ± 7.1° and 45.5° ± 10.0°, respectively. The IS group had larger PI, PT and SS but a smaller ST than the asymptomatic group (*P* < 0.05), while other sagittal parameters were similar (*P* > 0.05, Table 1). Though ST was significantly different between the two genders in the normal group (*P* < 0.05), other parameters were similar between males and females in either group (P > 0.05, Fig. 4).

**Table 1:** Comparison of demographic data, sagittal parameters and postural patterns

Items	Normal adults (*n* = 126)	Scoliotic adults (*n* = 117)	*P* value
Age (y)	26.4 ± 3.7 (26.0, 27.1)	26.3 ± 4.7 (25.4, 27.1)	0.777
BMI (kg/m^2^)	22.0 ± 3.2 (21.4, 22.6)	22.2 ± 3.8 (21.5, 22.8)	0.731
Sagittal spinopelvic parameters (mean ± SD; 95%CI)			
Thoracic kyphosis (°)	39.1 ± 11.0 (37.1, 41.0)	37.0 ± 15.0 (34.2, 39.7)	0.214
Lumbar lordosis (°)	54.0 ± 10.0 (52.3, 55.8)	54.6 ± 12.1 (52.4, 56.8)	0.703
Sacral slope (°)	37.2 ± 6.7 (36.0, 38.3)	39.2 ± 8.4 (37.6, 40.7)	0.039*
Pelvic incidence (°)	41.2 ± 7.1 (39.9, 42.4)	45.5 ± 10.0 (43.6, 47.3)	0.000*
Pelvic tilt (°)	4.5 ± 2.4 (4.1, 4.9)	7.9 ± 7.5 (6.5, 9.3)	0.000*
Spinosacral angle (°)	131.8 ± 6.9 (130.5, 133.0)	131.9 ± 8.9 (130.2, 133.5)	0.925
Spinal tilt (°)	94.6 ± 3.9 (93.9, 95.3)	92.7 ± 4.2 (91.9, 93.5)	0.000*
Sagittal postural patterns (*n*, percent)			
Roussouly 1	31 (24.6%)	25 (21.4%)	0.665
Roussouly 2	17 (13.5%)	20 (17.1%)	
Roussouly 3	62 (49.2%)	53 (45.3%)	
Roussouly 4	16 (12.7%)	19 (16.2%)	
C7-subgroups (*n*, percent)			
C7-anterior	6 (4.8%)	29 (24.8%)	0.000^#^
C7-posterior	120 (95.2%)	88 (75.2%)	

*BMI* stands for body mass index

*Statistically significant at *P* < 0.05, unpaired student *t* test (two-tailed)

#Statistically significant at *P* < 0.05, Pearson’s χ2 test (two-tailed)

**Table 2:** Demographic data and Cobb angles of coronal curves for the recruited IS adults

Curve types	*n*	Cobb angles of structured curves (mean ± SD; 95%CI))^a^		
		Proximal thoracic (°)	Main thoracic (°)	Thoracolumar/lumbar (°)
Lenke 1	43 (36.8%)	*NA*	47.8 ± 9.6 (44.8, 50.8)	*NA*
Lenke 2	24 (20.5%)	40.2 ± 8.8 (36.5, 44.0)	60.3 ± 12.1 (55.2, 65.4)	*NA*
Lenke 3	13 (11.1%)	*NA*	67.1 ± 15.7 (57.6, 76.6)	58.2 ± 19.9 (46.2, 70.2)
Lenke 4	16 (13.7%)	52.4 ± 7.9 (48.2, 56.5)	86.0 ± 10.5 (80.3, 91.6)	49.9 ± 6.9 (46.2, 53.6)
Lenke 5	13 (11.1%)	*NA*	*NA*	44.6 ± 4.7 (41.7, 47.4)
Lenke 6	8 (6.8%)	*NA*	38.9 ± 7.5 (32.6, 45.2)	61.2 ± 10.7 (52.2, 70.2)

*NA* stands for not applicable

^a^Only structured Cobb curves were included and presented

Normal and IS groups had a comparable distribution of Roussouly classification (*P* > 0.05), and Type 3 was the largest for both groups, found in 49.2% and 45.3% cases, respectively (Table 1). Though the distribution of Roussouly types was similar between the two normal genders, it was different between IS males and females (*P* < 0.05), with more females possessing a well-balanced sagittal standing posture, namely, Type 3 (Table 3).

**Table 3:** Comparison of the whole sagittal alignments between males and females

Items	Normal adults			Scoliotic adults		
	male (*n* = 76)	female(*n* = 50)	*P* value	male (*n* = 54)	female (*n* = 63)	*P* value
Sagittal postural patterns (*n*, percent)						
Roussouly 1	37 (23.7%)	23 (22.1%)	0.686	12 (20.3%)	13 (18.8%)	0.044*
Roussouly 2	28 (17.9%)	10 (9.6%)		9 (15.3%)	13 (18.8%)	
Roussouly 3	70 (44.9%)	54 (51.9%)		20 (33.9%)	38 (55.1%)	
Roussouly 4	21 (13.5%)	17 (16.3%)		18 (30.5%)	5 (7.2%)	
C7-subgroups (*n*, percent)						
C7-anterior	5 (6.6%)	1 (2.0%)	0.401	17 (34.5%)	12 (19.0%)	0.120
C7-posterior	71 (93.4%)	49 (98.0%)		37 (65.5%)	51 (81%)	

* Statistically significant at *P* < 0.05, Pearson’s χ2 test (two-tailed)

In all, 24.8% of IS adults belonged to the C7-anterior subgroup compared to 4.8% of normal adults (P < 0.05, Table 1). Males and females in both groups had a similar proportion of C7-anterior subgroup (P > 0.05, Table 3).

## Discussion

Roussouly classification has become a valuable approach for characterizing sagittal standing posture patterns. This classification system can outline four distinct types of sagittal profiles, and each type dominates in an age range, predisposes to different spinal pathologies and even indicates for a certain surgical strategy [1, 8–10, 12, 13]. Thus, it is beneficial for spinal surgeons to be aware of the difference of sagittal spinal balance between normal and IS populations. Since the values of sagittal parameters are affected by many factors, recruiting strategies must be meticulously planned to maximally maintain consistency of these factors, such as age and the stance during filming. The two groups in this study had similar age modalities (Table 1). Moreover, this study seemed immune to imaging-relevant influence, since the aforementioned radiographic protocol has been consistently conducted in our hospital.

This study found that the values of some sagittal parameters in IS patients deviated from the norm (Table 1). Three pelvic parameters, PI, PT and SS, significantly increased in IS patients. PI is an important pelvic parameter that can represent the potential of pelvic compensation for the spinal malalignment caused by some spinal pathologies [5, 12, 14–16]. Therefore, the increased PI and PT suggested a larger retroversion of the pelvis and thus demonstrated the existence of pelvic compensation. The pelvic compensation could influence the spinal alignment, considering the excellent linear correlation between PI and LL in the normal population, which was simply formulated by the eq. LL ≈ PI + 9° (±9°) [17–19]. This biomechanical compensation was basically effective, which was proven by the maintenance of the distribution of Roussouly types in the IS population compared with that of the norm (Table 1).

There are few previous articles exploring the sagittal standing postures in a scoliotic population. Yu et al. [20] reported that 42.5% of the Caucasian IS population belonged to Roussouly Type 3, but his study recruited both adolescents and young adults. Therefore, the result might deviate from either the exact proportion found in adolescent IS or adult IS, considering that some sagittal parameters, including PI, PT and LL, increase with age in the growing child [21, 22]. Hu et al. [6] reported that the proportion of Roussouly Type 3 in scoliotic adolescents was 42.4%, which is lower compared to the proportion of 45.3% in scoliotic adults found in the present study (Table 1). We speculate that this difference is age-associated. As a child ages, his/her pelvis gains pelvic retroversion, which can provide a higher compensatory capacity and prevents forward displacement of the gravity axis in the case of a spinal deformity such as scoliosis. Therefore, the proportion of the well-balanced sagittal standing posture, namely, Roussouly Type 3, is maintained.

This study showed IS adults had a higher SS than normal adults (Table 1). According to Roussouly’s reports [1], higher SS in IS adults should be accompanied with higher proportions of Roussouly Type 3 and Type 4 (Fig. 1). However, this study displayed a similar distribution of Roussouly types between IS and normal populations (Table 1). We speculated that this similarity was related to less coherence of lumbar alignment, another important determinant of Roussouly classification, to the increased PI and SS in the IS population [5, 15]. Another underlying reason in addition to PI-based regulation might be the complex inter-dependence of spinal three-dimensional malalignments in IS, which was presumably associated with the joint effects of “anterior overgrowth”, vertebral rotation and spinal buckling [2, 23, 24]. Therefore, the transition of the whole spine-pelvis complex in IS is more complex than that of the normal population. In other words, higher SS is not always accompanied with higher proportions of Roussouly Type 3 and Type 4 in the IS population but is comparable or has slightly lower proportions, as shown by the findings of this study (Table 1) given the joint effects of the aforementioned factors. However, the above conclusion is not definitive and needs further study for verification.

Asymptomatic males and females demonstrated a similar distribution of Roussouly types, whereas a significant difference existed between IS males and females (Table 3). In all, 55.1% of female IS subjects belonged to Type 3, which was higher than that of IS males. IS females were prone to standing neutrally, which was somehow unexpected since female IS patients tend to have an unfavourable prognosis [5]. Whether this divergence indicated any clinical advantage for IS females could not be arbitrarily asserted, especially in the absence of longitudinal follow-ups. This study also found that normal females had increased absolute values for all parameters except TK compared with normal males, although without statistical significance, corresponding to the previous studies in normal populations [4, 21, 22]. This means that normal females tended to possess prominent lordosis and/or kyphosis, which are usually seen in Roussouly Type 3 and Type 4. This might explain the finding of higher proportions of Type 3 and Type 4 in normal females than in males, which were 68.2% versus 58.4%, respectively (Table 3). However, this was not the case for IS males and females, in whom the total proportions of Type 3 and Type 4 were quite close (64.4% versus 62.3%), although IS females were predominantly Roussouly Type 3 (Table 3). This phenomenon might be related to the aforementioned ill-matched transition of spine-pelvis complex in IS adults. Correspondingly, we found that the divergence of sagittal parameters was more trivial between the two IS genders (Fig. 4), but this finding was not unique [6–8]. Therefore, the sagittal balance in the IS population was more complex and affected by the two related factors, namely, the inherent malalignment of this entity and the regulation of the PI-based system.

Mac-Thiong et al. [4] stated that the relative position of C7 plumbline with respect to the sacrum and HA was an important indicator for categorizing the global sagittal balance (Fig. 3). Generally, the forward displacement of the C7 plumbline was associated with a higher risk of developing spinal pathology [4, 9, 21]. This study found that 24.8% of the IS population stood with forward displacement of the C7 plumbline, which was significantly higher compared to 4.8% in asymptomatic adults (Table 1). In other words, the anterior shifting of the whole spine was more common in the IS population. This conclusion was reinforced by the finding of a lower ST in the IS population (Table 1). In addition, the finding that the proportion of the C7-anterior subgroup was similar between males and females in both groups implied that the occurrence of the forward displacement of the C7 plumbline might not be correlated with gender.

## Conclusions

In summary, this study was primarily dedicated to defining the deviation of sagittal standing postures in an IS population from the norm and found that there was no apparent correlation between specific Roussouly types and IS. However, there were some limitations. First, the source of the controls was restricted and was not from general society (see “Materials”), and these subjects had higher homogeneity in habits, lifestyle, etc., which might compromise the reliability of this study. Second, coronal deformities may affect the sagittal spinal profile, as previously reported by Hu et al. [6]. Thus, correction surgery has to restore both coronal and sagittal alignments. For example, low TK values occur in IS patients with thoracic curves, and so an effective correction of coronal curves should be accompanied with good restoration of TK. Secondary adaptation and adjustment of LL as well as pelvic sagittal parameters are then performed, and the overall sagittal balance is regained. The present study, however, mainly focused on the research of the deviation of the whole sagittal spinal balance in IS patients from the norm and did not include research and discussion regarding the effect of coronal deformity on the sagittal spinal profile.

## Abbreviations

CI: Confidence interval
HA: Hip axis
IS: Idiopathic scoliosis
LL: Lumbar lordosis
PA: Posteroanterior
PI: Pelvic incidence
PT: Pelvic tilt
SD: Standard deviation
SS: Sacral slope
SSA: Spinosacral angle
ST: Spinal tilt
TEM: Technical error of measurement
TK: Thoracic kyphosis
